# Explaining experiences of community-dwelling older adults with a pro-active comprehensive geriatric assessment program – a thorough evaluation by interviews

**DOI:** 10.1186/s12877-018-1025-7

**Published:** 2019-01-14

**Authors:** Wanda Rietkerk, Merel F. Smit, Klaske Wynia, Joris P. J. Slaets, Sytse U. Zuidema, Debby L. Gerritsen

**Affiliations:** 1 Department of General Practice and Elderly Care Medicine, University of Groningen, University Medical Center Groningen, Groningen, The Netherlands; 2 Department of Health Sciences, Community and Occupational Medicine, University of Groningen, University Medical Center Groningen, Groningen, The Netherlands; 3 Faculty of Medical Sciences, University of Groningen, University Medical Centre Groningen, Groningen, the Netherlands; 4 Leyden Academy on Vitality and Ageing, Leiden, The Netherlands; 5 Department of Primary and Community Care and Radboud Alzheimer Centre, Radboud University Medical Center, Radboud Institute for Health Sciences, Nijmegen, the Netherlands

**Keywords:** Frailty, Effectiveness, Satisfaction, Qualitative research, Improving care

## Abstract

**Introduction:**

Pro-active assessment programs are increasingly used to improve care for older adults. These programs include comprehensive geriatric tailored to individual patient preferences. Evidence for the effects of these programs on patient outcomes is nevertheless scarce or ambiguous. Explaining these dissatisfying results is difficult due to the multi-component nature of the programs. The objective of the current study was to explore and explain the experience of older adults participating in a pro-active assessment program, to help to clarify the effects.

**Methods:**

Semi-structured in-depth interviews were held with 25 participants of a pro-active assessment program for frail community-dwelling adults aged 65+. This study was part of an evaluation study on the effects of the program. Transcripts were analysed with thematic analysis and cross-case analysis.

**Results:**

The participants’ mean age was 78.5 (SD 6.9) and 56% was female. The majority of the participants were satisfied with the program but based this on communication aspects, since only a few of them expressed real program benefits. Participant experiences could be clustered in six themes: (1) All participants expressed the *need for a holistic view* which was covered in the program, (2) the *scope of the CGA* was broader than expected or unclear, (3) the program delivered un*expected* but valued *help*, (4) participants described a very low sense of *ownership*, (5) *timing* of the program implementation or the CGA was difficult and(6), participants and care workers had a different *view on what to consider as a problem*. These experiences could be explained by three program components: the degree of (the lack of) *integration of the program within usual care*, the *pro-active screening* method and the broader than expected, but appreciated *multi-domain approach*.

**Conclusion:**

Older adults’ need for a holistic view is covered by this outpatient assessment program. However, their engagement and the correct timing of the program are hampered by the pro-active recruitment and the limited integration of the program within existing care. Furthermore, satisfaction seems an insufficient guiding factor when evaluating CGA programs for older adults because it does not reflect the impact of the program.

**Electronic supplementary material:**

The online version of this article (10.1186/s12877-018-1025-7) contains supplementary material, which is available to authorized users.

## Background

Traditional ways of organizing health services are often unable to meet the heterogeneous needs of older adults with multi-morbidities [1]. Affordable solutions for organizing care are required to better meet the needs of older adults [2]. One way of adapting care to the needs of older adults is to organize person-centred and integrated care [2, 3].

One common element of integrated care is the Comprehensive Geriatric Assessment (CGA) [4]. A CGA is defined as “a multidimensional process to determine an elderly person’s medical, psychosocial, functional, and environmental resources and problems, linked with an overall plan of treatment and follow-up, to improve overall patient functioning and independence” [5]. The execution of programs offering CGA differs across settings, including community settings [6]. In a community setting, these programs pro-actively select a proportion of the home-dwelling older adult population. This is often based on frailty or multi-morbidity. These individuals are then offered a multi-domain CGA with the aim of longer independence and reduction in hospitalization and institutionalization.

These outpatient assessment programs have been extensively researched, however studies have shown inconsistent effects on clinical outcomes [3] and scarcely any effects on functional dependency [7]. This inconsistency in findings can be explained through the heterogeneity of these multi-component programs [8]. Different programs may encompass various combinations of potentially effective and ineffective components, with the latter masking benefits to different extents. Programs can differ in many aspects: overall program aim, domains focused on in the CGA, disciplines carrying out the CGA, as well as the level of influence over the recommendations given after the CGA and follow-up period. Studying the separate components of these programs, their implementation and how they fit together has been suggested [9] to optimally design programs that lead to improved patient outcomes [3, 10]).

One important perspective influencing the implementation and effects of programs is the consumer perspective [11] – in this case, the older adult. This perspective is often overlooked and is proven to be different from the most often used provider or organizational perspective [12]. Insight into the experience of older adults who have received a pro-active CGA program can help understand the relevance of program components and their coherence. Therefore the aim of this study is to explore and explain experiences of older adults who participated in a pro-active outpatient CGA program.

## Methods

As part of an evaluation study into the effects of the outpatient pro-active assessment program Sage-atAge, we conducted qualitative, semi-structured interviews into the experiences of older adults participating in an outpatient pro-active assessment program. We complied with the COREQ checklist in conducting and reporting this study [13]. For detailed information about the methodology and how COREQ requirements were met, see Additional file 1.

### Setting

The Sage-atAge program is an outpatient assessment program offered to home-dwelling older adults (65+) by seven general practices in a rural area in the north of the Netherlands. A postal questionnaire was distributed among 3004 older adults and completed by 1455 of them. This questionnaire captured frailty, care complexity and health-related issues. Frailty was assessed using the Groningen Frailty Indicator (GFI). GFI comprises 15 items, covering four domains: physical, social, cognitive and psychological. The total score ranges from 0 to 15; a higher score indicates higher level of frailty [14]. All older adults with a substantial frailty level (GFI > 2) were invited for a CGA (*n* = 708). The CGA consisted of a consultation with a geriatric nurse or elderly care physician [15] and focused on multiple domains (physical, functional, psychological, social and living). By protocol, the assessor was advised to extend the assessment with measurement instruments for psychological, social or functional needs. For example, when cognitive complaints or depressive feelings were expressed. Pharmacist and dental care worker assessments were also offered. A consult from an allied healthcare professional, such as a physiotherapist, dietitian or psychologist could be added to the CGA when deemed necessary by the nurse or physician. The consult aimed to reveal and formulate goals with the older adult in order to attain or preserve well-being. The healthcare workers involved were trained in using motivational interviewing - a method for encouraging people to make behavioural changes to improve health outcomes [16]. After the CGA, written recommendations were offered to the older adults and their general practitioners (GPs). The program’s effects on older adults’ morbidity and general well-being will be evaluated in a controlled before-after study. Alongside, a thorough process analysis is carried out. The current study on the experience of the older adults with the Sage-atAge program is part of this process analysis.

### Participants and recruitment

Older adults who had participated in the Sage-atAge program were invited to the interviews within four months after receiving the CGA. Between May 2015 and February 2016 older adults were purposively sampled to create a wide variation on demographics (age, sex and frailty level) and experience of diverse parts of the program (different care workers conducting the CGA). We planned for more than 20 interviews to provide enough “information power”. The sufficiency of the sample size was concluded from a diverse range of dimensions of our study: a small subject but with a heterogeneous sample, no predefined theory and a cross-case analysis technique [17]. We stopped interviewing when we reached data saturation.

Eligible older adults were sent an information letter by post about the purpose of the interview study and practical information about the interview. Within a week after receiving the letter, the interviewer (MS or WR) telephoned the older adult to check whether they were interested and an appointment was scheduled when they expressed an interest.

### Data collection

The topic list (see Additional file 2) was prepared by WR and MS, discussed with SU and DG and tested in 25 pilot interviews which were not part of this study. It consisted of the following subjects: 1) recall of and experience with the program and the CGA, 2) recall and opinion regarding the recommendations provided, 3) motivation for participation in the program, 4) goals and disabilities in life and 5) experience with health care workers in general. The topic list was adjusted following a few interviews and a discussion of the findings: the view of the participants on healthy ageing was investigated to improve our understanding of participant coping strategies.

Semi-structured interviews with open-ended questions were held, following the River Structure, i.e. personal experiences of a participant could lead to a new head course, so not all questions from the topic list would necessarily be used in all interviews.

Interviews were performed by MS (medical student) and WR (elderly care physician in training) who were trained in interviewing. Before starting the interview, participants were reassured that the transcripts would be anonymized and their health care providers would not be able to trace back any opinions to individual participants. This was done to encourage participants to express their own opinions. When the interviewer’s medical background was known participants appeared to compare their health care workers with the interviewer. Therefore, in the final 15 interviews, the medical background of the interviewer was not revealed. The latter may have led to fewer ´desirable´ answers being provided [18, 19]. All interviews were audio-taped and transcribed verbatim.

### Data analysis

This study aimed to both explore, as well as explain the experience of older adults [20, 21]. Therefore we used thematic analysis [22] with a focus on a cross-case analysis using a constant comparative method [17, 23].

Transcripts were open-coded by two researchers (WR & MS). Experiences were explored first and allocated to themes. Subsequently, experiences were matched to program components in order to provide an explanation for the experiences. Thematic analysis was conducted in thorough steps: a within-case analysis was carried out first to cross-check the interpretation of the older adults’ stories and experiences between the researchers. Once subthemes emerged the focus was shifted to an inter-case analysis in which these themes were compared. This enabled a multidimensional typology to be drawn from the older adults’ potential viewpoints of the CGA program. The observations and themes were regularly discussed by three researchers (WR, MS, DG). The coherence and connections between themes were visualized multiple times in a coding tree and network view, and then discussed within the whole research team (WR, MS, DG, JS, KW, SZ). The literature was extensively searched to help explain and understand relationships between themes.

### Data presentation

**Fig. 1:**
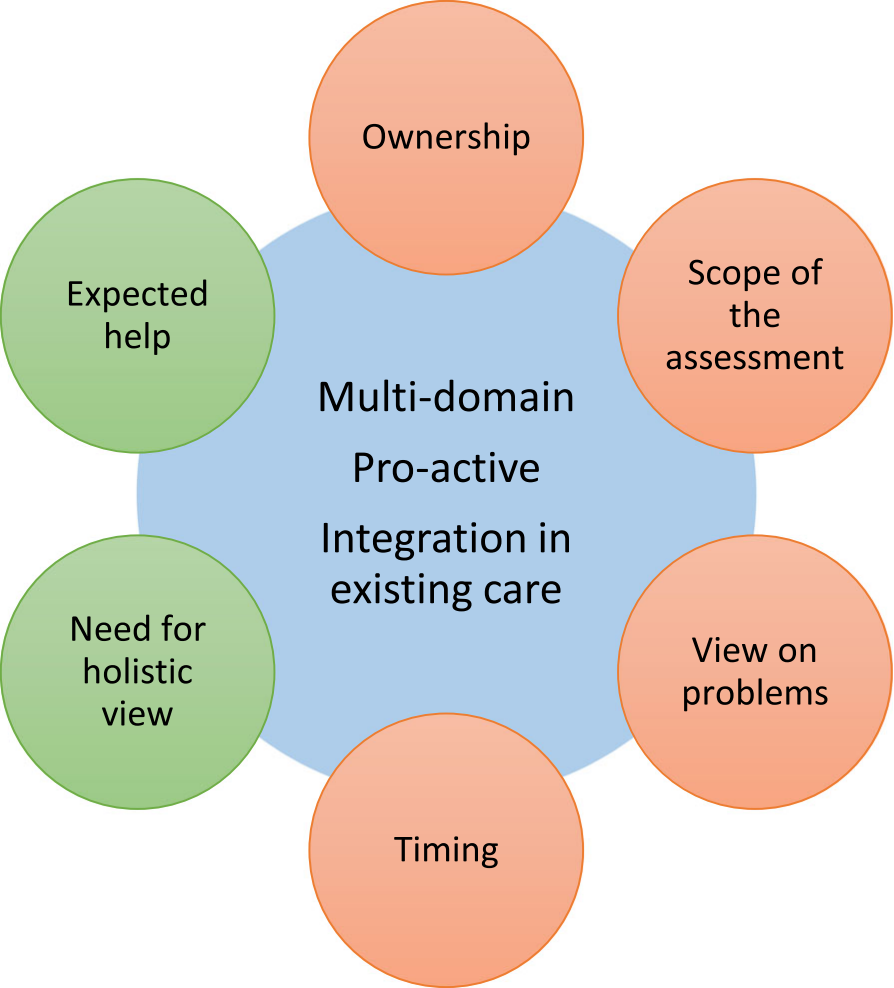
Themes encompassing the experience of outpatient assessment program participants, relevant program components and their coherence. The small circles forming the outer layer are representing the themes encompassing the experience of participants of an outpatient assessment program. The big inner circle contains the relevant program components. The coherence of the program components and the experiences are marked by the colour of the small circles. The red small circles indicate that themes are negatively influenced by the program components and the green small circles indicate that themes are positively influenced by the program components.

Individual details of the participants and their CGA were listed in Table 1. Ages have been presented in ranges and recommendations have not been described into detail in order to the minimize the risk of patient identification. Participants have been numbered successively. The identified themes have been described and an illustrative primary quote is provided with every theme in the Results section to support and clarify themes. Secondary quotes were gathered per theme. These have been listed separately in Table 2 to improve readability and accessibility of the results [24]. The number and gender of the participants has been provided with every quote. These are referred to with successive codes (e.a. A1). All quotes have been translated into English by a professional translator. Finally, the relationship between relevant themes is visualized in Fig. 1.

**Table 1:** Demographic and program characteristics of the interviewed older adults (*N* = 25) who participated in Sage-atAge

	Demographics					Intervention characteristics CGA by			
ID	Gender	Age range	Marital status	Educational level^a^	GFI^b^	initial professional	additional assessment	CGA at	Domain of Recommendations
1	M	95–100	married	medium	5	Nurse	Physiotherapist	Home	physical (risk of falling)
2	M	85–90	married	medium	6	Nurse/Ph/D		Centre	functional, dental, (vision)
3	F	80–85	married	medium	8	ECP		Home	physical, psychological (fitness)
4	M	80–85	widower	medium	11	ECP		Home	physical, social (lung condition)
5	M	75–80	married	medium	7	ECP		Home	NA
6	F	75–80	married	medium	3	Nurse/Ph/D		Centre	physical, dental (fitness)
7	M	80–85	married (to P8)	medium	6	Nurse/Ph		Centre	physical, social (leisure activities)
8	F	80–85	married (to P7)	low	8	Nurse/Ph		Centre	physical, social (side-effects)
9	F	85–90	divorced	low	4	Nurse/Ph		Centre	physical, psychological, medication (coping)
10	F	65–70	married	medium	6	Nurse/Ph		Centre	physical, dental (fitness)
11	M	70–75	widower	high	5	Nurse		Home	physical, psychological (depressed mood)
12	M	75–80	married	high	6	Nurse/Ph/D		Centre	physical (fitness)
13	F	80–85	widow	medium	6	Nurse		Home	NA
14	F	65–70	widow	low	5	Nurse/Ph/D		Centre	functional, medication (vision)
15	M	75–80	married	high	4	Nurse		Home	NA
16	M	80–85	married	low	3	Nurse/Ph		Centre	physical, psychological, social, medication (coping)
17	F	80–85	married	medium	3	Nurse/Ph/D		Centre	physical, psychological, dental (pain)
18	F	70–75	divorced	low	7	Nurse/Ph		Centre	physical, social (leisure activities)
19	F	75–80	married	low	6	ECP		Home	physical, functional, medication (pain)
20	F	85–90	widow	low	5	Nurse/Ph		Centre	psychological (coping strategies)
21	M	75–80	married	medium	3	Nurse	Psychologist	Centre	functional, psychological (care giver support)
22	F	75–80	married	medium	8	Nurse/Ph/D		Home	psychological, functional, social (vision)
23	M	70–75	married (to P24)	medium	9	ECP	Physiotherapist	Home	physical, functional, social (leisure activities)
24	F	65–70	married (to P25)	medium	5	Nurse/Ph		Centre	psychological (coping with family problems), dental
25	F	75–80	married	medium	10	ECP		Home	physical (physiotherapy), psychological (care giver support), social, living (home adaptation)

*M* male, *F* female, *ECP* elderly care physician, *Ph* pharmacist medication review, *D* dental care worker, *Y* yes, *N* no. *NA* not applicable

^a^low = primary school (or less) or lower vocational training; medium = secondary school/vocational training; high = Higher vocational training or university

^b^GFI = Groningen Frailty Indicator (range 0–15), a higher score indicates more frail

**Table 2:** Overview of sub-themes supporting all main themes and secondary quotes grounding the subthemes

Main themes	Supporting subthemes	Relevant secondary quote
Experience		
	Recall and understanding of the intervention	A1 “The GP has a lot of elderly people in his practice, do they all get a letter? I: Yes, they do. P: So everyone ... so not that you say we will pick out a few ...? I: Yes {} But you tell me that you did not receive the invitation [to fill in the questionnaire] ... P: No, I did not get it. I: What kind of invitation did you receive? P: Well, just an invitation to come here [to the centre], that was the only invitation I got.” P9 F A2: “Because I think you had to turn to the GP as well, didn’t you? The GP had then indicated which people were eligible for this. So in that respect the doctor played a part in it, didn’t she? So that’s, well, yes, people do have a lot of illnesses or whatever, right? In that way, it’s been brought into action you might say, hasn’t it? But otherwise she has nothing to do with it, I think. She probably won’t have time for that ...” P6 F
	Satisfaction is not about the effect of the program	B1 “I have been treated nicely. Otherwise, I can’t say anything negatively about it. I wouldn’t advise against it to someone else either, but recommend it, oh well, I don’t know. Anyway, there’s nothing that I wasn’t happy with. That you say like, well, I would rather not have done it. {but} I don’t know what’s in it – there’s nothing in it. Not for me.” P21 M
Need for a holistic view		
	Appreciation with the broad view of the program	C1 “My blood was also just checked again this week, because they doubted the thyroid gland, which was only slightly on the edge [the doctor] thought. Then, I went to be checked again, I’ll just wait for [that result] again. You can be very tired of that, too. But then again, we have been very tired for a very long time. And then [the nurse from Sage-atAge] said: “But that’s only logical, woman, you have so much going on in your head, that alone should get you tired”. I think: well, you are right. That was true enough.” P24 F
	Other care workers are not meeting this need…	C2 “but I hope that they [care workers] can do something quickly. You always hope for that and yes, also in hospital. I don’t get anything there either. [there they say]: “You may come back in a year”, just like that. They just don’t give a moment’s thought to anything.” P4 M
	…and they are not expected to meet this need	C3 “we only visit the GP when it’s very much needed, right? If you ... really have problems ... Or, yes, real problems ... If you’re really ill, say, then you’ll visit the doctor.” P6 F
	Participants experience a lack of interest into this need with other care workers	C4 “Sometimes I also notice that with GPs: They just listen to your heart for a moment: “Oh yes, it’s still beating.” And then they listen to your lungs for a moment, “yes they are also still working. Well now, so you are not dead.” And for the rest, you may just figure it out. So no feeling with the human being behind the patient at all. [The GP can’t take care of everything] He doesn’t have to, but he should have an antenna for picking up someone’s signals.” P11 M
	Need for support	C5 “I don’t have the opportunity to always read everything I’d like to [because of vision problems]. {} I don’t play a part in anything anymore, do I? I listen to the radio to hear the news all day long, and if something is wrong, well ... But, there are also things you should just actually read, shouldn’t you? So that it really sinks in. {} [with the nurse at Sage-atAge] I could at least just tell my story and I thought that in itself this was a start to set everything in motion, wasn’t it?” P2 M C6 “Especially checking the medication is important to me as well. And nothing had to be changed about it, but that people paid attention to it. You can never know.” P2 M
Scope of the CGA		
	Unable to recall the agenda of the CGA	D1 “I: And do you still remember what [the CGA] was about then? P: Yes, it was also all about those ordinary things. Yes, I just call it ordinary things. It was all about how you lived and what you could still do and this and that and about all of those things. But exactly, the specific details, that I don’t know anymore.” P20 F D2 “I: Yes ... And when the doctor came here to visit ... Do you still remember how that was then? {} P: Yes, I do. Talking a bit about everything, right? S: Yes, of course you start with an open mind, don’t you? I: And that conversation ... How did that go? P: Well, it went alright. Yes, I think I could give an answer to whatever she asked.” P5 M
	Uncertainty about the goal of the program	D3: “I: And before the doctor came here - did you have any idea of what she would come and do here? P: No, not at all, right? No, because we thought it was something that our doctor would help with or so. Yes, that’s what I was thinking. And am I right? That she will then have a better overview of our family or something?” P3 F
	Questionnaire guided the agenda of the CGA	D4 “I: Had you then thought in advance about what you were going to discuss during that conversation? P: No, I hadn’t, because that had already been noted in my questionnaire, right? [The conversation] was more an explanation of what I had already said in the questionnaire. Well, she asked some additional questions about and around that and so on. So, well, I felt that it was going quite alright.” P11 M D5 “S: At some point [the nurse] then says: “It’s about time that I should deal with my questions, because otherwise it will take much too long.” I: because what actually were her questions then? P: Well those were, they actually were related to the list that I had filled in. And so I did answer those.” P15M
	Scientific design of the program	D6 “Ok, well, that conversation was not useless. But yes, I actually did, I thought, answer all sorts of questions in the questionnaires, so I believe that conversation didn’t have any added value. That was not this lady’s [Sage-atAge nurse] fault, but let’s put it this way, I’m not any the wiser. Well, it was a research, so then you are not supposed to be any the wiser, but you are expected to make the researcher wiser.” P12 M
Expected help		
	Unexpected problems discussed	E1 “I’ve also received a card from her, because it was also about some personal things with her in the end, and that was very nice, too, and well, then she had something like, then give – I’ll give you my card, right, if ever you think you’ll need me again, you may always call me.” P24 F
	Unexpected solutions	E2 “I did speak with someone from social support. They now know what the situation is like here, so in general I benefited from it to some extent. If anything happens to me, they know about my wife’s situation [for whom he is care giver]..” P21 M
Ownership		
	Passive role	F1 “They really want you to. That’s why I say: I’ll just take part in it. {} For my doctor and for myself as well, of course.” P9 F F2 “There’s no harm in it anyway. I thought: They are launching a new project there, I’ll just contribute to that. But not with a certain expectation or so.” P6 F
	Initiation	F3 “Yes, she would discuss it with the GP ... And then you don’t hear anything. Then you have to ask about it yourself. {} You would like to contribute alright, but I think the other side should come up with something as well.” P5 M
	Agenda	F4 “you’re waiting for what that lady would say.” P1 M F5 “I: But do you still remember what you talked to her about? P: Yes, also about, these things rather. Yes, yes, but everything specifically, she asked and then I just answered in fact. That’s how you should see it.” P4 M
	Actions expected by the care workers	F6 “I: So the care worker came to visit you at home. P: Yes, he did, because I could have come myself [to the research centre]. But that was not necessary.” R23 M F7 “So I knew that [the nurse] would contact the GP. But I didn’t know what would happen next, so then I already thought, yes, should I be the one to take initiative, will I have to call her later or how does that work. Oh well, I thought, just wait and see for a while. But then that [family doctor] visited, personally. {} The GP had actually signed me up to that project, so it’s only logical that people from that project will give feedback to the GP about the results.” P11 M
	No actions carried out by participant	F8 “That’s how I found out that the cause was the diuretics that were affecting me badly. So then I said to that pharmacist like, what do you say about this? Shall I just leave them? Because I still have some problems with dizziness… Never heard of anymore.” P8 M F9: [reading out the goal card]: “‘Increasing the activities around movement a bit and possibly go to [the community centre]’. You have to do that apparently because we haven’t heard anything from that either. Actually we haven’t heard about anything at all.” P7 F
	Unsolved misconceptions	F10 “Yes, we would like to contribute, but we didn’t really know what it means. And I actually still don’t know, but I thought it was about help, for the doctor. For our family doctor, and that she would ... would then explore our household a bit and what was there.” P3 F F11 “I never really understood that it was for me. I had the idea that it was part of the research.” P12 M
Timing		
	Ageing is about uncertainty	G1 “I: [how can we make sure we reach those who will benefit from this]? P: Well, of course at this age that may change per month, eh? So yes, that’s difficult.” P7 M
	Changes occurred within timeframe of program	G2 “then .. the first time someone came here, nothing was wrong with me, but then there was during the second time.” P3 M G3 “I have very bad eyesight, and then [the Sage-atAge nurse] also talked about Visio [a vision-aid centre]. And then I said to her like, “Well, I won’t need that yet. I’ll be fine like this.” But now I do need them. {} It used to be fine, until four, five weeks ago. I suddenly got a dark spot in front of the eye {} Yes, I needed it faster than I expected myself.” P14 F G4 “And I also have a sore knee and I didn’t mention that {} at that moment it wasn’t hurting so much and then I forgot about it.” P10 F
	Synchronization with other health care	G6 “I: Has that also been discussed then [at the CGA]? P: No, it hasn’t ... That, eh, I haven’t mentioned that anymore. Because we were already working on that [with the GP].” P2 M G7 “I: Have you also discussed the memory with [the Sage-atAge nurse]? P: No, I haven’t. {} Then we didn’t know it yet. Then we hadn’t visited that doctor in hospital ...” P16 M
	Counselor would solve timing difficulty	G8 “{Sage-atAge} is a start I think, yes. Well, this is only just an inventory. {} I: and would you like it if that nurse would see you again? P: Well, it doesn’t necessarily have to be a nurse, because there is nothing to nurse here. So it doesn’t really matter who that is as long as he’s part of such a project or organization. {} I: and that he will come back once in a while? P: Yes, otherwise it doesn’t make sense. A one-off doesn’t make sense. So that should actually become standard procedure.” P11 M G9 “All those people [he met at a ward when he was hospitalized] could use a director. I think that would be a good addition to supporting ill people, including simple material matters. {} There are so many annoying things in life, which will be going to be 100 %, one thousand percent more difficult if you fall ill. {} that if they have a question about something, that they know they may call someone in confidence.” P12 M
View on problems		
	Questionnaire is lacking the narrative	H1 “[the Sage-atAge nurse] said: “You sometimes feel lonely, too.” I said “No, not that I know of.”. She said “You did fill that in.” I said “Well, then that was a mistake.” So therefore she has been here again and we talked about it once more. {} But yes, I’m on my own, but I don’t feel lonely.” P14 F H2 “Alright, so there may have been a few leads for [the Sage-atAge nurse] to come here, because I might have answered a bit differently from the average answer, that’s possible. I was probably a doubtful case.” P15 M
	Expecting physical scope	H3 [reads out problem on the goal card]:“‘preferably be a bit more mobile’. Yes, I do fortunately have my car, but otherwise I would be completely stuck at home! [reads out] ‘Preferably be a bit more among people’ ..., oh well, I am. {} No, I can’t do all that much with this [goal card]. [I only have a problem] with diabetes, which isn’t mentioned on it. I: and the things that are on it, are these matters for you that were relevant at that point? P: Yes, that’s private, if I want to play cards then I’ll just do so. Which, it seems to me, doesn’t have anything to do with that. S: That is a leisure activity.” P8 F H4 “Well, we went through everything a bit. I am quite reasonably aware of how I’m put together. It isn’t an examination, not a medical examination. So like ingrown toenails and so on, they are not mentioned.” P12 M
	No urgency for prevention	H5 “But anyway, yes, you should try to live a bit healthily, but not at all costs. Because then I think the quality of life is losing out. Then you do have a healthy body that may want to get old, but a certain quality of life is part of it as well, and I think that is missing then. If I can’t smoke my cigarette, can’t have my drink, yes, then nothing will be left anymore.” P11 M H6 “I have to exercise more but that is considerably inhibited by my heart condition {}. I can’t do more than that. Things should remain pleasant, right? I think it’s important to get “healthily old” but that’s not an end in itself. You have to be able to grow old in a pleasant way. There’s no point at all in filling your days with horizontal bar exercises in order to win another year.” P12 M
	Coping/Secondary control/Acceptance	H7 “We aren’t getting old in a healthy way. When we get older, everything starts to crack, I sometimes say. But yes, you hear that from a lot of people {} Yes, they all suffer from it in some way.” P14 F H8 “And I also try to walk in succession as far and as long as possible. Because sometimes I don’t have any energy left. Then I walk a short distance and then I have to sit on my walker. And then walk a bit further. And then when I have walked all the way out and back, then I praise myself. I think that’s so beautiful then. I did manage to walk all that. That used to be quite normal, but now everything isn’t normal.” P20 F
	Unfounded hope	H9 “I: [What did you expect from Sage-atAge?] R: Well, I … that it could be useful to me when they could help me with this [with the oxygen therapy] {} But anyhow, I don’t get any support, I don’t have to count on that, no.” P4 M

Corresponding code (e.a. A1) “*quote”* Participant number, Sex (Female/Male). *S* spouse, *I* interviewer, *P* participant. {}: text left out to increase readability. []: text added or paraphrased to increase readability

## Results

### Participants

Twenty-five participants were interviewed about 2.5 months (range 1.5–5.5) after the CGA for 60 min on average (range 30–106). Almost all interviews were held at the participants’ home; sometimes with the spouse present. Table 1 lists the characteristics of each participant showing a broad range of frailty levels, ages ranging from 67 to 95; almost half of them were married and more than half of them had a low educational level.

When asked, all participants were able to remember their experiences with Sage-atAge; sometimes after being assisted with prompts, except for one woman. She had recently moved to a residential home and could not recall the CGA nor completing the questionnaire (P13).

### Exploring the experience of participants

None of the participants could recall the separate parts of the program and their coherence: the questionnaires, the CGA, the recommendations for them and their GPs and their own responsibility in these components.

*“I: what was the Sage-@Age program to you? P: an inventory of how the GP works, whether I am satisfied with the general practice, that was what I understood from it.” P25 F.*

For additional citations for all described themes, see Table 2.

All of the participants expressed satisfaction when referring to the program. Further analysis suggested that levels of satisfaction related to communication with the CGA workers, and not about the value of the program (B1).

*“I immediately felt comfortable with her. I could speak with her in an easy way. You were able to ask anything. That was very good. {} I sat there and had a very nice feeling afterwards and was not nervous or anything at all, not at all. {} Oh well, nothing else has happened, I mean I have asked questions, she has asked questions ... other than that nothing special.” P9 F*

Further examination of the participants’ experience of the program, revealed six main themes.

#### Need for a holistic view

Participants appreciated the CGA and questionnaires for their focus on a broad view on their lives and health complaints (C1).

*“Because it has to do with being seen. That you really see the other person as a whole individual. That you are not just that pelvis, or that arm that is broken, or whatever, but that you see the human being. That is the most important thing for me. I: and how do you notice that you are being seen? P: looking at someone, not directly behind the computer, but making eye contact, I think that's very important. And that you also feel that someone is listening. That you are not just an ailment that needs to be resolved. But that you are seen as a human being.” P25 F*

Some participants had not discussed these aspects previously with other health care workers (C2). They expected that their own GP or medical specialist would not have time to discuss these aspects (C3), or they experienced a lack of interest from doctors in their problems (C4). Participants considered the CGA as reassuring in terms of the fears they experienced in daily life (C5, C6).

*“You could talk about anything, she was very attentive and - perhaps that is the most important – just the very idea that she said “Well, if something is wrong you can always call me”. {} I think that is the most important thing. That you know that you are not on your own.” P6 F.*

#### Scope of the CGA

The scope of the CGA seemed unclear for the participants. They did not know what the CGA would be about beforehand, and they could not recall clearly what it had been about afterwards (D1, D2).

“*I: what did you expect in advance of the appointment? P: just a conversation about the complaints we had written on [the questionnaire] and about how we looked to the future: continue to live here, to live independently. But I actually did not have an image of how that would be. Well I answered her questions, and apart from that we just talked a bit.” P24 F*

Though participants expected it to be broader than a consultation at the GP, they were unsure what to discuss within a CGA (D3). The program’s questionnaire seemed to be a barrier to bringing up new – more important – topics. (D4, D5).

*“I: Did you have any questions for her? R: No. What could I ask someone like that? I don’t know. {}. Well, she just asked questions about the paper we filled in.” P19 F*

This can partly be explained by participants thinking the scientific goals of the project were more important than discussing their own problems (D6).

*“I went into this process with the understanding of “you have been picked out. Would you like to participate in the study?” So then I thought okay, that's fine, I want to, not with the preconceived goal that they had to do something for me, or whatever, that developed in the course of the conversation.” P25 F*

#### Expected help

Problems were discussed within the program for which the older adults had not yet sought or found help. This could result in unexpected revelations of (E1) and even to unexpected solutions to problems (E2).

*“I went to [the ophthalmologist] and then they said “We can’t do anything for you anymore”. After two operations, on both eyes. {} {Then the Sage-atAge nurse advised to go to a vision-aid centre}. {} Then I thought, well, isn’t this something. You go to [the hospital], and they did not know what to do with me.” P22 F.*

#### Ownership

Almost all participants described their own role in the program as passive (F1, F2). They explained their role as ‘wait and see’, because the initiative for the assessment was not their own (F3). They tended to wait for the care workers to bring about subjects during the CGA (F4, F5).

*“Well, I didn’t know that [topics of conversation], you don’t know those beforehand, right? I had to indicate what I had. So, that’s what I did. I’ve just done what they asked of me.” P8 F*

Participants waited for Sage-atAge to schedule appointments when and whenever this was deemed necessary (F6). They expected the GP to be informed by the CGA nurse or physician (F7). Afterwards, they tended to wait for the GP to contact them about the advice given:

*“And she [Sage-atAge nurse] told me what I already knew: {} “At some point I’ll report to your GP how I see things.” So, I assume that the report is with the GP. I have not been to the GP since this spring. And he has not been here either, so he probably won’t have discovered anything disturbing.” P15 M*

Participants almost never acted themselves whenever the expected action had not been carried out by the GP and CGA care workers (F8, F9). Furthermore, participants tended not to resolve their own questions regarding the program design (F10, F11).

*“I: Will you see [the Sage-atAge nurse] again? P: Well, that wouldn’t be bad. And it may well be that we get another call, from this Sage with Age. It could be. {} I don’t know how it continues. No idea, but there might be a follow-up, or maybe they will ask in a year's time how it is going.” P24*

#### Timing

Participants stressed that timing of the program implementation or the CGA was difficult. They attributed this to the fluctuation of symptoms in time and concomitant health concerns (G1). The timing of the program components were therefore delicately balanced in three ways. For instance, occasionally symptoms had already changed or resolved in the time period between completing the questionnaires and attending the CGA (G2). Secondly, sometimes just after the CGA some important deterioration took place or the symptoms occurred only outside of the CGA (G3, G4, G5).

*“I have not heard anything yet [no invitation for a CGA] I had also completed [the questionnaire] but then after that time I got that arrhythmia which I didn’t have before. I didn’t have much at the time and then all of a sudden there is something, right?” Spouse of P5 who was not invited to the CGA because of the absence of frailty on the self-assessment questionnaire.*

Additionally, when symptoms had already been covered in other health appointments (or were to be in the future) this seemed to be a barrier for discussion during the CGA (G6, G7). Multiple participants advocated a personal counsellor for this timing difficulty. The latter was described as someone who could be contacted spontaneously with no prior planning for a broader range of problems than those considered appropriate for a GP, for example for reassurance or practical help (G8, G9, G10).

*“Though I might need someone next week, or this week. But then I have to make an appointment during office hours again. And I find that difficult. I would just like to have someone whom I could call and say to them ‘Hey, I would like to get something off my chest’ or ‘Could you lend an ear to me’. I need that most and I find that with my friends {} but you know, I sometimes want something outside my circle of friends.” P25 F*

#### View on problems

There also seemed to be a difference between the participants and the care workers in the definition of problems. The participants differed in four ways in their view on problems highlighted by the questionnaire or care worker. Firstly, they objected to problems revealed by the questionnaire (H1, H2). Secondly, it was sometimes questioned by participants whether problems on domains outside of the physical domain were part of the program scope. This meant these issues would not be expected to be seen as a relevant problem to discuss (H3, H4). Thirdly, participants seemed to stress less importance on preventive actions than care workers. (H5, H6). Finally, sometimes symptoms had been a burden some time ago and were therefore highlighted in the questionnaire, yet as participants had already adapted to them, it no longer seemed important to discuss them in the CGA (H7, H8).

*“Just gradually you start to put things into perspective again and then everything becomes a bit more common again. First you have to process it and then I think like: gosh we have so much, we have healthy children, we have healthy grandchildren, what more could you wish for? Life is not endless, we [all] have to leave here in the end.” P6 F*

Multiple participants described problems that were overlooked by the program (both by questionnaire and care workers) in addition to the difficulty of signalling problems with the questionnaire. These problems were also neglected or not solved by care workers from existing care. They were hoping the program would offer a solution for these problems, but were also disappointed that the program was not able to address these problems. (H9, H10).

*“Because sometimes there are a lot [problems]: because I always have a buzzing in my ear. They don’t look further into it. And I thought, well maybe something will result from it, that's why I thought it was fine. I: You had hoped that they might investigate the ear? P: Yes, everything actually. I have a swollen hand and swollen foot, I have pains in my legs every night. {} I went to the hospital several times, [they say:] “well there is nothing wrong with you”. Well, I thought they’ll look at it thoroughly and that was what I thought of the assessment. {} Yes, I really looked at it from a completely different point of view, let’s be honest.” P18*

These six themes explored the participants’ experience. When trying to explain the experience, the themes coalesced with three program components (See Fig. 1).

### Explaining the participant experience - program components

The participant experience, was considered in light of the program design in order to provide an explanation for these experiences. We identified three program components which seemed to have a part in the participants’ experience.

#### Multi-domain approach

Firstly, the program’s multi-domain approach attended to the *need for a holistic view* that was expressed by the older adults. Secondly, it may, however, also be used to explain the participants’ confusion about the *scope of the CGA*. Thirdly, it could be a reason for the difference in the *view on problems*: the explanation why the care worker would focus more on prevention than the participant appreciated.

#### Pro- active

Another program component which seemed to influence the experience was the pro-active approach. The program was pro-actively offered to a(n older adult) population. This meant that the steer towards the CGA was led by answers on the screening self-assessment, more specifically, whether these answers complied with the CGA inclusion criteria. This is contrast to a consult or appointment where patients seek help themselves or are individually referred by their GP. This component had the positive effect of delivering un*expected help*. Problems were discussed which had not involved a care worker to-date and where care was not expected (as yet). The pro-active induction had two drawbacks though. Firstly, it was often experienced as wrongly *timed* because of the dynamic of symptoms related to ageing. Secondly, it seemed to amplify the passive role of participants in the program: they experienced a lack of *ownership* with regard to the topics to discuss and in terms of adhering to advice.

#### Integration with existing care

The third program component which seemed to influence the experience of older adults was the one-off aspect of the program and the way it was incorporated in the existing care processes. Due to the lack of *ownership* and the uncertainty about *the scope of the CGA* older adults did not prepare for the CGA and did not actively engage in the agenda during the CGA. Afterwards they sometimes felt important problems had not been discussed. The single contact with the CGA care worker provided a one-time opportunity only, which also caused friction with the *timing* of the CGA. Reaching consensus on the *view on problems* in this single contact was also troublesome. The lack of integration in the existing care structure was a barrier to implementing care after the CGA,. Because of the lack of *ownership* by the older adults, they did not carry out recommendations by themselves and waited for their GP.

## Discussion

In this article we described the experience of older adults with an outpatient assessment program and aimed to explain the coherence between this experience and program components. Although older adults expressed satisfaction this did not cover their whole experience with the program; they also expressed a lack of ownership in the program, experienced problems with the timing of the program, were uncertain about the scope of the CGA and their views on problems seemed to differ with the care workers. Importantly, the program seemed to address their need for a holistic view and delivered unexpected help.. In aiming to explain this broad range of experiences we found coherence with three program components: multi-domain approach, pro-active sampling and integration in usual care. By connecting the experiences to the program components we gained insight into potentially relevant factors for improving care for older adults.

### Embedding in literature

Similar to our findings, Darby et al. reported that satisfaction applied to the contact with the geriatrician within an in-patient CGA program, but it also appeared concurrently with a lack of understanding regarding the meaning of the intervention [25]. For out-patient CGA programs both with and without subsequent interventions, the discrepancy between satisfaction and efficacy has been underlined before [26]. It is also noted in other settings, like residential care [27] and within the concept of person-centred care [28]. Notwithstanding this complicated nature of satisfaction, satisfaction (with care) is still used as an outcome measure of CGA evaluation studies, e.g. by Ekerstad et al. [29]. However, there is also a trend towards the evaluation of ‘experienced care’ instead of satisfaction [30]. Our results emphasize the importance of this development for evaluating experiences of health services.

The participants appreciated the multi-domain approach of the program as they expressed a need for a holistic view. This need is in line with other research into the experience of older adults with regular health care [31] and integrated care [32]. However, the holistic view of the CGA also hampered the participants to get a grasp on the scope of the CGA. This could be explained by the expectations of patients that interaction with care workers would mainly focus on physical complaints: the Voice of Medicine [33]. Another problem with the broad scope of the CGA is the dilemma in *the view on problems* between older adults and care workers. Extensive literature exists on the change in priorities given to problems experienced when ageing, [34] the declining need for primary prevention and goals being reset [35, 36]. This all underlines the difficulty of really tuning in on the needs and preferences of older adults.

This program, as most outpatient assessment programs, had a preventive strategy design. Our data showed that the advantage of pro-actively delivered care was the experience of unexpected help. This decreases patient delay: participants were not seeking help for their needs yet because they did not expect any result from a consult with their GP or medical specialist. Reasons provided by the literature for not seeking help for problems have attributed these problems to age [37] or being unaware of possible solutions [38].

The difficulty of the pro-active strategy was the sub-optimal timing of the CGA that was experienced by participants. When disabling arises within the process of ageing, it is characterized by multiple and possibly interrelated disability episodes [39]. Van Houtum et al. noted that patients only have an increased need for support when they experience progression or deterioration of their disease, and care workers should be able to adjust their timing to the course of disease [40].

Another finding linked to the pro-active design was the lack of ownership the participants expressed. Lack of ownership is known to decrease commitment to goals and the chance of attaining goals [41], decrease self-management ability [42] and having a negative impact on effect [43]. It is noteworthy that being a patient in itself seems to cause a passive attitude towards care workers and their disease-management [44].

The care of Sage-atAge lacked proper integration with existing health and social care. The negative impact of this solitary aspect and (therefore) lack of control of the implementation of recommendations on the effect of a CGA was described in various research. In a long-standing meta-analysis, solitary CGA programs showed no effect [45], in contrast to home-visiting programs with follow-up visits or programs embedded in general practice [6, 46]. Kagan et al. demonstrated that a lower integration within primary care is linked to lower adherence to recommendations [47]. An explanation for this connection could be that GP need support in acting upon CGA recommendations [26]. Despite the overwhelming amount of literature about the importance of integration of care and the difficulty of implementing this collaborative way of working [48] there is still not enough attention to embedding in existing care or to the context in which outpatient programs are carried out [9].

The consequence of the solitary nature of the intervention is amplified by the CGA being a solitary consult lacking follow-up. For appropriate recommendations, adapted to the goals and needs of the older adult, more than one consult is commonly needed, as has been demonstrated in the shared decision making literature [49, 50].

### Strengths and limitations and further research

The qualitative design of this paper allowed us to reveal a coherence between experiences and program components, possibly explaining contradictory findings in intervention studies within CGA-research, and a broader insight than a survey would have provided. Two drawbacks of this qualitative study are nonetheless worth noting. Firstly, during the interviews participants repeatedly postulated a link between the interviewer and the Sage-atAge care workers. This could have introduced socially desirable answers. Nevertheless, numerous remarks and complaints about the conduction of the intervention were still voiced.

Secondly, the findings might not be generalizable to outpatient assessment programs or CGAs with a different design than Sage-atAge, for example those with only one or two of the program components discussed. Further research could study these components separately in different CGA settings considering their effects on participant experiences and health outcomes.

When the screening instrument revealed a ‘problem’ older adults were reluctant to considering this as a problem, even though they had marked the response on the questionnaire. There is possibly a discrepancy between the problems flagged by the questionnaire and the way older adults experience these problems. Questionnaires are increasingly used to guide care (pathways). Hence, more research is needed into these possible discrepancies.

## Conclusion

An outpatient assessment program fits into person-centred care, as it is able to meet the older adults’ need for a holistic view. Next to that, with its’ pro-active approach it is able to deliver unexpected help to some of the participants. However, the correct timing and engagement of older adults is hampered by pro-active recruitment and limited integration of the program within existing care. More attention needs to be paid to these program components and implementation strategies when designing and evaluating pro-active and person-centred care for older adults.

Therefore, there seems to be a need for unscheduled availability of care workers working holistically and integrated within the standard health care of older adults. This was suggested by our participants and has been concluded in other research into the older adults’ perspective for improving standard care [51, 52].

This study underlines that satisfaction seems an insufficient guiding factor when evaluating care programs for older adults as it appears to have no link with the experienced effects of the assessment program. Conversely, three program elements appeared to be of importance for explaining this experience: The multi-domain scope, the pro-active approach and the integration with existing care. These factors should be addressed when developing outpatient assessment programs and evaluation studies.

## Additional files

Additional file 1: Detailed applied methodology following the consolidated criteria for reporting qualitative studies (COREQ) 32-item checklist. (DOCX 29 kb)

Additional file 2: Topic list for semi-structured in-depth interview with Sage-atAge participants. (DOCX 22 kb)

## Abbreviations

CGA: Comprehensive Geriatric Assessment
COREQ: Consolidated criteria for Reporting Qualitative studies
GFI: Groningen Frailty Indicator
GP: General Practitioner
