# Supplementary material for: Explaining experiences of community-dwelling older adults with a pro-active comprehensive geriatric assessment program – a thorough evaluation by interviews
Source: BMC Geriatr. 2019 Jan 14;19:12. doi: 10.1186/s12877-018-1025-7 (PMC6332689; doi:10.1186/s12877-018-1025-7)
Supplement: Supplementary file 1 — Detailed applied methodology following the consolidated criteria for reporting qualitative studies (COREQ) 32-item checklist. (DOCX 29 kb) [file 12877_2018_1025_MOESM1_ESM.docx]

Additional file 1 - Detailed applied methodology following the consolidated criteria for reporting qualitative studies (COREQ) 32-item checklist

| **No. Item** | **Guide questions/description** | **In our research:** |
| --- | --- | --- |
| **Domain 1: Research team and reﬂexivity** | |  |
| *Personal Characteristics* | |  |
| 1. Interviewer/facilitator | Which authors conducted the interview? | MS/WR |
| 2. Credentials | What were the researcher’s credentials? | MS: BSc. WR: MD |
| 3. Occupation | What was their occupation at the time of the study? | MS: Master student. WR: Phd student |
| 4. Gender | Was the researcher male or female? | Female |
| 5. Experience and training | What experience or training did the researcher have? | MS: Medicine  WR: Medicine. Elderly Care physician  Research team:  The researchers have diverse backgrounds; medical (SZ, JS), health sciences (KW) and psychological (DG) and are all specialized in elder care. |
| *Relationship with participants* | |  |
| 6. Relationship established | Was a relationship established prior to study commencement? | The Sage-atAge program is initiated by an older adult care organization. Researchers carrying out the scientific evaluation are not explicitly involved in the program development and implementation.  MS twice observed the CGA of an participant before interviewing. She did not know the care workers.  WR knew the care workers and gave them training-on-the-job. She had no acquaintances with study participants before the interview. |
| 7. Participant knowledge of the interviewer | What did the participants know about the researcher? e.g. personal goals, reasons for doing the research | They were aware of the reasons for doing the research as part of the evaluation of the Sage-atAge program. In the invitational letter it was also noted that the researcher was a medical students (MS)  In the latest interview rounds, respondents were not informed about the medical profession of the interviewer (WR) which helped elicit more critical views on health care providers. |
| 8. Interviewer characteristics | What characteristics were reported about the inter viewer/facilitator? e.g. Bias, assumptions, reasons and interests in the research topic | It was written in the invitational letter and orally stated that the researchers had no conflict of interest with the outcome of the conducted CGA |
| **Domain 2: study design** | |  |
| *Theoretical framework* | |  |
| 9. Methodological orientation and Theory | What methodological orientation was stated to underpin the study? e.g. grounded theory, discourse analysis, ethnography, phenomenology, content analysis | Thematic analysis (1), cross-case analysis |
| *Participant selection* |  |  |
| 10. Sampling | How were participants selected? e.g. purposive, convenience, consecutive, snowball | By conducting purposive sampling, a sample of older adults was created with variation in their care complexity and experience of diverse parts of the program (i.e. assessment by nurse, elderly care physician, location of assessment at home or in the centre, additional assessment by pharmacist and follow-up visits).  Participants were invited 1-3 months after CGA. This time range was thought to warrant older adults still remembering details of the assessments, and would have had time to act on their formulated goals. The latest two samples were invited shorter after their assessment to improve recall of details of the communication with the care worker. |
| 11. Method of approach | How were participants approached? e.g. face-to-face, telephone, mail, email | Four sets of a maximum of ten older adults were selected, to make sure no additional participants would be invited after achieving data saturation.    Participants first received an explication letter in which the invitation by telephone was announced. Within a week they were contacted by telephone to ask for consent and – when consented – an appointment was made. |
| 12. Sample size | How many participants were in the study? | 25 |
| 13. Non-participation | How many people refused to participate or dropped out? Reasons? | In total, 42 older adults were invited of whom 27 consented to be interviewed. Reasons for not participating were severe illness (6), no apprehension with the research purpose (3), did not receive the assessment (1), no interest (1), died (2), not available by phone (2). One participant was not at home at the appointment and not available by phone afterwards and one withdrew before the interview took place. |
| *Setting* |  |  |
| 14. Setting of data collection | Where was the data collected? e.g. home, clinic, workplace | All interviews were carried out at home except for one, in which the participant preferred to come to the intervention center. She preferred speaking alone (without her spouse for whom she was the informal care giver) |
| 15. Presence of non-participants | Was anyone else present besides the participants and researchers? | Yes, a spouse was present in 14 of the 25 interviews. Two of the spouses were participants themselves and were interviewed afterwards (interviews 8 and 24). Present spouses were instructed not to respond except when asked for. |
| 16. Description of sample | What are the important characteristics of the sample? e.g. demographic data, date | See baseline table |
| *Data collection* |  |  |
| 17. Interview guide | Were questions, prompts, guides provided by the authors? Was it pilot tested? | A semi-structured interview guide was used, most often only to check at the end of the interview whether all topics were discussed.  The interview topic list was pilot tested by 2 students who carried out a pilot study among 25 participants. This pilot study focused on the experienced effects after Sage-atAge. After this pilot study, the topic list was adapted for the current study: the description of the program Sage-atAge in the participants’ own words was added to the topics, to improve our insight in the understanding of the participants with regarding the program design.  Prompts used during the interview were: pictures of the care worker who carried out the CGA, the invitation letter of the CGA and the goalcard.(2) |
| 18. Repeat interviews | Were repeat interviews carried out? If yes, how many? | No |
| 19. Audio/visual recording | Did the research use audio or visual recording to collect the data? | All interviews were audio-taped and transcribed (by medical students MS, ED and SAR) using the same instructions.  All transcripts were checked against the tape recordings (WR). |
| 20. Field notes | Were ﬁeld notes made during and/or after the interview or focus group? | During the interview, prompts were used to improve recall of the assessment and the program (i.e. picture of the care worker, questionnaires, goal card, invitational letter of the GP). After the interview, field notes were written and together with a summary added to the transcripts. |
| 21. Duration | What was the duration of the interviews? | Mean duration was 64 minutes, with a range from 30 to 106 minutes |
| 22. Data saturation | Was data saturation discussed? | The research group discussed findings and data saturation multiple times. Data saturation was reached after 25 interviews. This is mentioned in the manuscript as well |
| 23. Transcripts returned | Were transcripts returned to participants for comment and/or correction? | Receiving a summary of the interviews was offered after every interview, only one participant approved to this. She did a minor suggestion after returning the summary. |
| **Domain 3: analysis and ﬁndings** | |  |
| *Data analysis* |  |  |
| 24. Number of data coders | How many data coders coded the data? | WR, MS, DG |
| 25. Description of the coding tree | Did authors provide a description of the coding tree? | No |
| 26. Derivation of themes | Were themes identiﬁed in advance or derived from the data? | Themes were derived from data |
| 27. Software | What software, if applicable, was used to manage the data? | Analysis was done with Atlas.ti 7.5.15 (Atlas.ti Scientific Software Development, Berlin, Germany). |
| 28. Participant checking | Did participants provide feedback on the ﬁndings? | See 23. |
| *Reporting* |  |  |
| 29. Quotations presented | Were participant quotations presented to illustrate the themes/ﬁndings? Was each quotation identiﬁed? e.g. participant number | Yes, see Results section and Table 2 |
| 30. Data and ﬁndings consistent | Was there consistency between the data presented and the ﬁndings? | Yes |
| 31. Clarity of major themes | Were major themes clearly presented in the ﬁndings? | Yes, see Results section and Figure 1 |
| 32. Clarity of minor themes | Is there a description of diverse cases or discussion of minor themes? | Yes |

1. Braun V, Clarke V. Using thematic analysis in psychology. Qual Res Psychol [Internet]. Taylor & Francis Group; 2006 Jul 21 [cited 2015 Nov 5];3(May 2015):77–101. Available from: http://www.tandfonline.com/doi/abs/10.1191/1478088706qp063oa

2. DiCicco-Bloom B, Crabtree BF. The qualitative research interview. Med Educ. 2006;40(4):314–21.
