# Supplementary material for: Explaining experiences of community-dwelling older adults with a pro-active comprehensive geriatric assessment program – a thorough evaluation by interviews
Source: BMC Geriatr. 2019 Jan 14;19:12. doi: 10.1186/s12877-018-1025-7 (PMC6332689; doi:10.1186/s12877-018-1025-7)
Supplement: Supplementary file 2 — Topic list for semi-structured in-depth interview with Sage-atAge participants. (DOCX 22 kb) [file 12877_2018_1025_MOESM2_ESM.docx]

Additional file 2 – Topic list for semi-structured in-depth interview with Sage-atAge participants

| **Topic** | **Sub-topic** | **Interview questions** | **Additional sub-questions** |
| --- | --- | --- | --- |
| **Experience** | Experience | **How do you recall your experience at Sage-atAge?** | What components of the program do you recall?  Prompts used: picture of the professional who performed the assessment and/or self-assessment questionnaire used for screening for eligibility and/or location of the assessment centre was described.  Did you discuss the program with your relatives? |
|  | Expectation | What did you expect from the assessment? Did you had topics in mind to discuss? | Why was this (not) discussed? What made this happen? Or what did you need to discuss this? |
| **Communication (quality)** | Trust and confidentiality | You did not know [nurse] beforehand… | What made you feel (un)comfortable? |
|  | Quality of goals and recommendations | Were goals formulated or recommendations given at the assessment? | Did you receive a goal card? And what was on the goal card?  If applicable, a goal card was used as a prompt  What did you do with these recommendations and goal card? Did you discuss it with your GP/relatives |
| **Needs** | Added value of (a program like) Sage-atAge | What makes you feel better? | What bothers you in daily live?  Can you describe an ordinary day? |
|  |  | How can needs of older adults preferably be addressed? | How would ideal care for you look like? What can you/ your GP / other relevant care professional do to help to reach this? |
|  |  | What kind of needs do you have? | What did you discuss recently with your GP? Was this also discussed in the assessment? Why (not)?  What kind of help do you need? Was this dependency discussed in the assessment? |
|  | Cope with age  Healthy ageing | What does ageing imply for you?  What does healthy ageing mean to you? | What questions do you have for your GP? Can you discuss this with your GP? Why (not)? Does this bother you?  How do you take care of your own health? What decisions do you make about your lifestyle? How do you know what is best for you? |
| **Involvement** | Self-management preference | Can you describe a recent consultation with your GP (or other care professional involved)?... | What do you discuss with your children, friends, neighbours about health? What kind of advice do you give to them? |
|  | Disease-management preference | …and what happened after this consultation? | Who do you expect to take the lead? Why? |

GP: general practitioner
